# Acute kidney injury following cardiac surgery: current understanding and future directions

**DOI:** 10.1186/s13054-016-1352-z

**Published:** 2016-07-04

**Authors:** Jason B. O’Neal, Andrew D. Shaw, Frederic T. Billings

**Affiliations:** Department of Anesthesiology, Vanderbilt University Medical Center, Nashville, TN USA

**Keywords:** Cardiac surgery, Acute kidney injury, Renal failure, Cardiopulmonary bypass, Extracorporeal circulation, Hypoperfusion, Inflammation, Pigment nephropathy, Intravenous fluid management

## Abstract

Acute kidney injury (AKI) complicates recovery from cardiac surgery in up to 30 % of patients, injures and impairs the function of the brain, lungs, and gut, and places patients at a 5-fold increased risk of death during hospitalization. Renal ischemia, reperfusion, inflammation, hemolysis, oxidative stress, cholesterol emboli, and toxins contribute to the development and progression of AKI. Preventive strategies are limited, but current evidence supports maintenance of renal perfusion and intravascular volume while avoiding venous congestion, administration of balanced salt as opposed to high-chloride intravenous fluids, and the avoidance or limitation of cardiopulmonary bypass exposure. AKI that requires renal replacement therapy occurs in 2–5 % of patients following cardiac surgery and is associated with 50 % mortality. For those who recover from renal replacement therapy or even mild AKI, progression to chronic kidney disease in the ensuing months and years is more likely than for those who do not develop AKI. Cardiac surgery continues to be a popular clinical model to evaluate novel therapeutics, off-label use of existing medications, and nonpharmacologic treatments for AKI, since cardiac surgery is fairly common, typically elective, provides a relatively standardized insult, and patients remain hospitalized and monitored following surgery. More efficient and time-sensitive methods to diagnose AKI are imperative to reduce this negative outcome. The discovery and validation of renal damage biomarkers should in time supplant creatinine-based criteria for the clinical diagnosis of AKI.

## Background

Risk factors for acute kidney injury (AKI) are common among patients undergoing cardiac surgery (Table 1) [1], and partially explain why AKI occurs in up to 30 % of patients [2]. Many of these factors are not modifiable, such as advanced age, hypertension, hyperlipidemia, and peripheral vascular disease [3]. Other factors are specific to anesthetic, surgical, and ICU management, and physicians should be cognizant of these factors in order to eliminate or mitigate their effects. The unique characteristics of cardiac surgery, including cardiopulmonary bypass (CPB), aorta cross-clamping, high rates and volumes of exogenous blood product transfusion, and high doses of exogenous vasopressors, increase the risk of AKI compared with noncardiac surgery. These factors alter renal perfusion, induce cycles of ischemia and reperfusion, increase oxidative damage, and increase renal and systemic inflammation—all mechanisms implicated in the development of AKI [4].

**Table 1:** Risk factors for the development of acute kidney injury following cardiac surgery

Preoperative	Intraoperative	Postoperative
Advanced age	Complex surgery	Vasopressor exposure
Female gender	Cardiopulmonary bypass (CPB) duration	Inotrope exposure
Hypertension		Diuretic exposure
Hyperlipidemia	Need to return to CPB	Blood transfusion
Chronic kidney disease	Low hematocrit during CPB	Anemia
Liver disease	Aortic cross-clamp time	Hypovolemia
Peripheral vascular disease	Hypoperfusion	Venous congestion
Previous stroke	Hypovolemia	Cardiogenic shock
Smoking history	Venous congestion	
Diabetes	Emboli (cholesterol and other)	
Anemia	Inotrope exposure	

Strategies to prevent AKI are integral to the routine management of cardiac surgery patients. For example, intravenous fluid management, surgical and extracorporeal circulation techniques, and hemodynamic stability affect AKI development. Pharmacologic and many nonpharmacologic treatments have largely failed to reduce cardiac surgery-associated AKI in clinical trials, although some treatments may be effective in specific patients. Recent trials of high-dose perioperative atorvastatin and remote ischemic preconditioning (RIPC) therapy unfortunately have provided minimal evidence that these treatments reduce AKI following cardiac surgery [5].

When prevention fails, a prompt diagnosis of AKI is required to allow physicians to implement the few strategies that are known to improve renal function. The diagnosis of AKI typically includes the use of serum creatinine (SCr) concentrations and urine output. Urine output is relatively nonspecific, and increases in SCr concentration require several days, extending the time to diagnose AKI and initiate treatment. Measurement of urinary markers of kidney damage may provide a more rapid diagnosis, although candidate biomarkers require further validation before clinicians will incorporate them into routine patient care and expert groups implement them into AKI diagnostic criteria [6].

## Review

### Pathophysiology

**Fig. 1:**
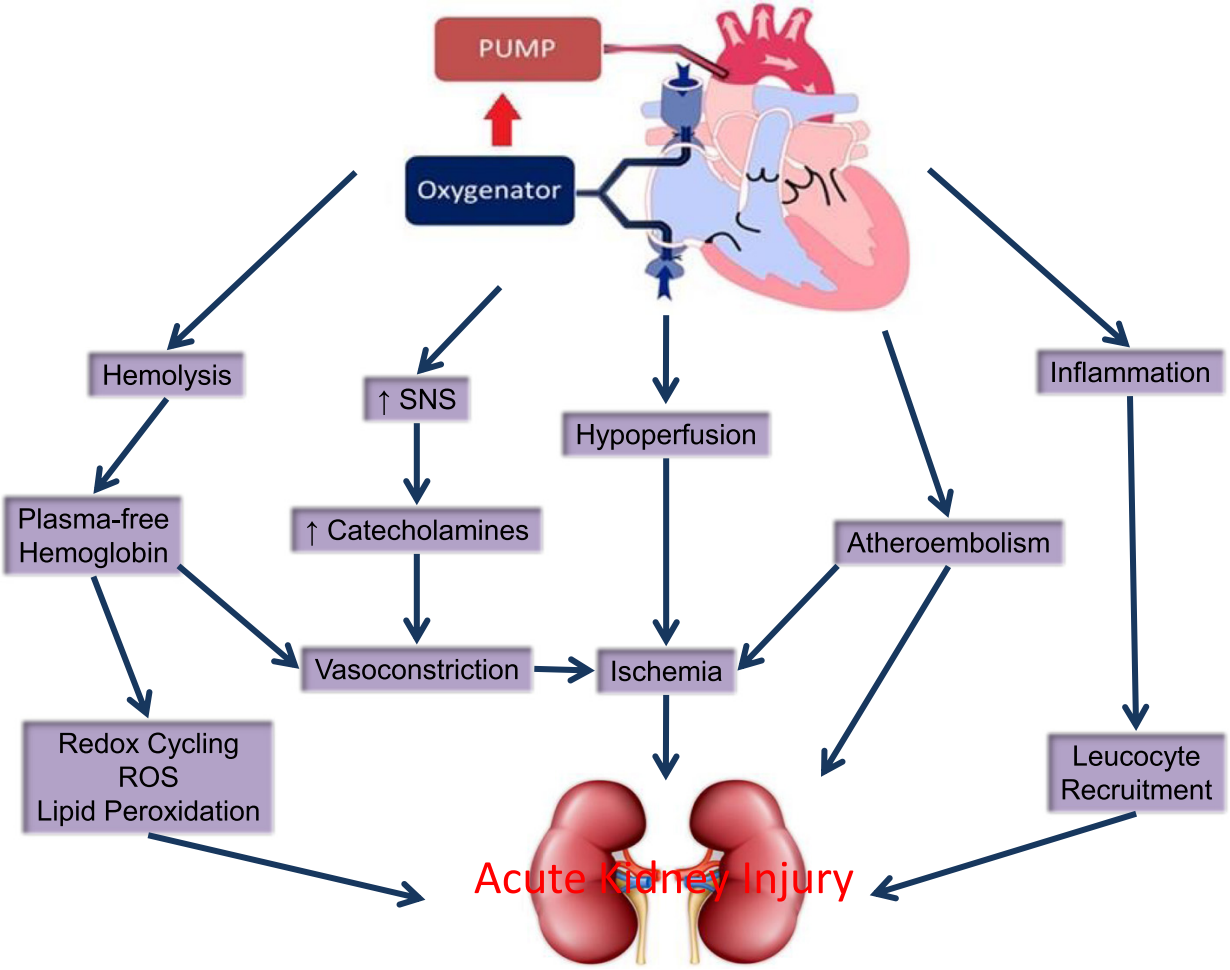
Pathophysiology of acute kidney injury following cardiac surgery. *SNS* sympathetic nervous system, *ROS* reactive oxygen species

Mechanisms of cardiac surgery-associated AKI include perioperative renal ischemia, reperfusion injury, CPB-induced hemolysis and pigment nephropathy [7], oxidative stress [8], and inflammation (Fig. 1).

Renal perfusion is complex and highly regulated. Although 20 % of cardiac output perfuses the kidneys, the majority of blood filtered by cortex glomeruli is shunted away from the vasa recta. This shunt may help maintain the electrolyte and water concentration gradients in the renal medulla required for tubule and collecting system reabsorption, but renders the renal medulla and corticomedullary junction hypoxic relative to other tissues (PO_2_ 10–20 mmHg) [9]. This may be a protective mechanism for oxidative injury but increases susceptibility to ischemia. During surgery many factors alter renal perfusion, and tubules at the corticomedullary junction and in the medulla are often damaged.

CPB provides nonpulsatile blood flow and may dysregulate the balance between cortical and medullary perfusion. Paradoxically, increased cortical perfusion may precipitate corticomedullary ischemia due to increased medullary oxygen consumption from increased solute transport [10]. Aorta cannulation and cross-clamping increase atheroemboli to the kidneys, further exacerbating ischemia and inducing inflammation [11]. Other factors including sympathetic nervous system activation, the endogenous release of circulating catecholamines, and induction of the renin–angiotensin–aldosterone cascade may further impair renal oxygenation during surgery [12–14].

Cardiac surgery also induces renal and systemic inflammation. Elevated postoperative plasma concentrations of inflammatory cytokines are associated with a subsequent diagnosis of AKI and increased mortality [15]. The mechanisms that increase inflammation during cardiac surgery are not fully understood, but contact activation from the exposure of blood to the CPB circuit, ischemia reperfusion injury, and oxidative damage all contribute [12, 16]. For example, ischemia and most notably reperfusion induce reactive oxygen species production [17, 18], and reactive oxygen species induce inflammation by upregulation of proinflammatory transcription factors, including nuclear factor kappa-B [19, 20]. Cytokines and chemokines recruit neutrophils, macrophages, and lymphocytes into the renal parenchyma. Parenchymal infiltration and activation of these immune cells increases AKI and leads to fibrosis [16].

**Fig. 2:**
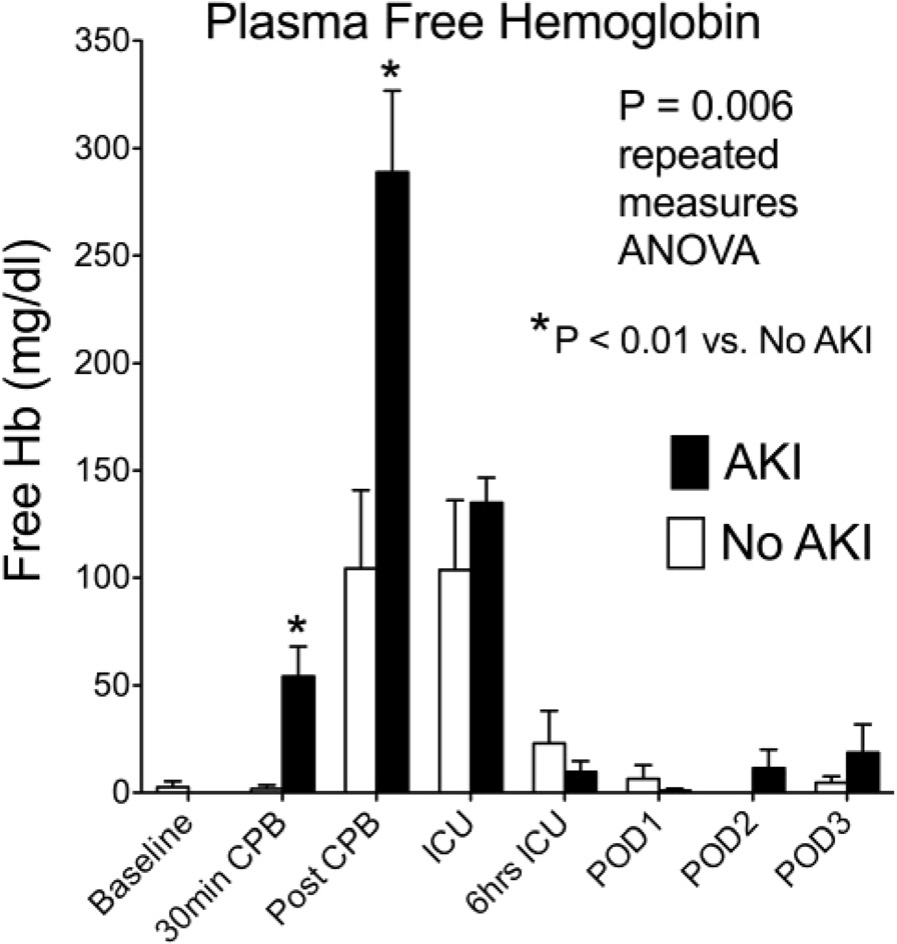
Perioperative concentrations of plasma-free hemoglobin (*Hb*) in acute kidney injury (*AKI*) and risk-matched control patients. Hb concentrations at baseline, 30 minutes into cardiopulmonary bypass (*CPB*), immediately following CPB, at ICU admission, 6 hours after ICU admission, and on the mornings of postoperative days (*POD*) 1, 2, and 3 in patients who developed AKI and in risk-matched (including identical CPB times) control patients who did not develop AKI. Postoperative AKI was associated with higher circulating concentration of free Hb during and immediately following CPB (*P* < 0.01) and throughout the study period (*P* = 0.006)

The CPB circuit contains a pump, oxygenator, suction catheters, and filters that damage erythrocytes and increase plasma-free hemoglobin [21]. Free hemoglobin depletes circulating haptoglobin and injures the kidneys by catalyzing free-radical production, precipitating with Tamms Horsfall proteins in the renal collecting system, and inducing renal arteriole vasoconstriction by eliminating nitric oxide [22–24]. In addition, circulating labile iron further increases reactive oxygen species production via the Fenton and Haber Weis reactions [25, 26], particularly in tissues where free hemoglobin and iron are sequestered, namely the kidney. In a case–control study of AKI patients and risk-matched controls, patients who developed AKI had twice the plasma-free hemoglobin at the end of CPB than those who did not develop AKI, despite similar AKI risk profiles and identical CPB durations in each group (Fig. 2) [27]. These data suggest that hemolysis and high concentrations of plasma-free hemoglobin, through induction of subsequent injurious mechanisms or from direct effects, may contribute to the development of AKI following cardiac surgery.

### Diagnosis

Establishing an accurate and timely diagnosis of AKI enables prompt treatment. Current consensus guidelines for AKI diagnosis use Kidney Disease Improving Global Outcomes (KDIGO) criteria [6]. KDIGO criteria define AKI as a 0.3 mg/dl (≥26.5 mol/l) SCr increase from baseline within 48 hours of surgery, a 50 % SCr increase from baseline within 7 days of surgery, or a decrease in urine output below 0.5 ml/kg/hour for 6 hours. All recent consensus criteria for AKI diagnosis, including RIFLE, AKIN, and KDIGO, use changes in SCr concentrations and urine output [6, 28]. Oliguria is common following cardiac surgery and typically occurs prior to SCr increase following renal injury, but often is an appropriate response to intravascular hypovolemia. For this reason and because accurate hourly urine output documentation is frequently poor, AKI is most often diagnosed by clinicians using SCr measurements. In a heterogeneous cohort of ICU patients, however, AKI using urine output criteria was common (60 % of the cohort) and associated with similar poor outcomes (dialysis and mortality) to AKI by SCr criteria alone [29]. AKI that met oliguria and creatinine threshold criteria in this study was associated with significantly worse outcomes than AKI from oliguria or creatinine alone. These results need replicating in cardiac surgery cohorts. Creatinine endpoints are better validated than oliguria endpoints but unfortunately require many hours or days to diagnose AKI after a renal insult and may be insensitive to mild kidney injury since the kidney can maintain glomerular filtration as nephrons are damaged. Conversely, small changes in SCr may reflect an ongoing systemic inflammatory process nonspecific to AKI. Nonetheless, the 0.3 mg/dl threshold is strongly associated with poor outcomes [30, 31]. What remains unclear is whether poor outcomes associated with stage 1 AKI result from the AKI or from an independent systemic process associated with the AKI.

Scientists have identified proteins released from the kidney during injury and others filtered by the kidneys that more closely reflect glomerular filtration. These markers of renal damage (e.g., NGAL, KIM 1, IL-18, NAG, and GST) and function (cystatin C) offer several theoretical advantages over SCr. Concentrations of damage biomarkers increase in the plasma and urine within hours of injury, may be more specific, and are more sensitive. Haase et al. [32] validated these benefits for NGAL when they demonstrated that NGAL-positive but creatinine-negative AKI independently predicted the duration of ICU and hospital length of stay. Further validation of these damage and function markers versus short-term and long-term clinical outcomes including dialysis, length of stay, mortality, and progression of chronic kidney disease (CKD) remains insufficient. These markers are most frequently judged relative to SCr-based AKI criteria, and SCr-based criteria provide a poor standard due to the sensitivity and specificity concerns related to renal injury outlined in the preceding paragraph. For this reason (limited validation) and others (limited availability and reimbursement), the aforementioned biomarkers are confined to research purposes and are not used in clinical practice today. The FDA recently approved the measurement of urinary tissue inhibitor of metalloproteinases (TIMP) 2 and insulin-like growth factor–binding protein (IGFBP) 7, markers of growth phase cell-cycle arrest, to aid in the risk assessment for moderate or severe AKI within 12 hours of cardiac surgery. Clinical use of these markers, marketed as Nephrocheck®, to predict AKI has been limited, and their validation versus acute and more persistent renal injury is ongoing. The ability to measure AKI damage markers in routine clinical practice, however, may help the renal community advance past the use of SCr to diagnose, predict, prognosticate, or monitor AKI.

Just as intensivists and nephrologists developed consensus criteria for AKI diagnosis, clinician scientists are hoping to adopt unified criteria for persistent renal injury. This will enable therapeutic, clinical practice, and epidemiologic data to be compared against a consistent benchmark – the same benefits AKI consensus criteria brought to the AKI field. The Major Adverse Kidney Events (MAKE) composite, consisting of a 25 % or greater eGFR reduction, dialysis, or death, meets this objective. Because some physicians believe AKI is transient and not a clinically relevant endpoint, the adaptation of MAKE as a primary endpoint may advance the field [33]. MAKE are often assessed at 30, 90, or 365 days after surgery. MAKE90 may be the best time point, because 90 days is a threshold that nephrologists use to declare AKI has progressed to CKD.

### Predicting risk

Accurate prediction of AKI provides the opportunity for clinicians to optimize high-risk patients, increase monitoring, enroll patients in clinical trials, and initiate preventative and therapeutic treatments. Several risk stratification systems exist for cardiac surgery patients. The best-validated scores predict severe AKI requiring dialysis and include the Cleveland Clinic Score and the Mehta Score [34, 35]. These scores use similar risk factors to predict AKI, although the Cleveland Clinic score offers the best discrimination [36].

Prediction of mild and moderate AKI is also important [37]. As such, Birnie et al. [38] analyzed data collected prospectively from over 30,000 subjects undergoing cardiac surgery at three hospitals in the UK to develop a model using KDIGO criteria for predicting all stages of AKI. The model’s risk prediction score for any stage of AKI (AUC, 0.74 (95 % CI: 0.72–0.76)) demonstrated better discrimination compared with the Cleveland Clinic Score and equivalent discrimination to the Mehta score. This is the first predictive model for all stages of AKI.

Incorporation of novel markers into prediction algorithms may provide additional opportunities to identify patients at high risk. To date, most studies have evaluated the ability of damage and functional markers to predict AKI as compared with clinical risk factors, but to our knowledge these have yet to be incorporated into prediction scores for AKI following cardiac surgery.

### Prevention

Several practical interventions are available for the prevention of AKI, starting with the management of intravenous fluid administration. Although cardiac surgery is associated with microvasculature injury and tissue edema [39], colloid administration is not clearly superior to crystalloid. Colloid administration marginally improves maintenance of intravascular volume compared with crystalloid [40], and both albumin administration and hydroxyethyl starch administration have been associated with increased rates of AKI in some studies [41, 42]. However, a recent clinical trial of hypoalbuminemic patients undergoing off-pump coronary artery bypass surgery (OpCAB) demonstrated that albumin administration decreased the incidence of AKI compared with crystalloid [43]. The results suggest hypoalbuminemia may be a modifiable risk factor for AKI associated with OpCAB. It is not clear whether benefit was derived from improved renal perfusion or direct effects of albumin on the kidneys [44]. Additional studies are needed, particularly in on-pump surgery where inflammation and tissue edema are often worse than in off-pump surgery. With regard to crystalloid composition, isotonic normal saline administration has recently been associated with AKI, possibly as a result of the excess chloride load [40, 45]. A meta-analysis of 21 studies that included 6253 subjects found high-chloride solution administration to be independently associated with increased risk of AKI (relative risk (RR), 1.64 (95 % CI: 1.27–2.13); *P* < 0.001) and metabolic acidosis (RR, 2.87 (95 % CI: 1.95–4.21); *P* < 0.001), although mortality was unaffected [46]. Interventional trials of hyperchloremic and balanced-salt crystalloid solutions have thus far confirmed that administration of normal saline may be harmful or at least no different from administration of a balanced-salt solution during critical illness [47, 48].

“Goal-directed therapy” incorporates specific monitoring techniques to guide intravenous fluid and vasoactive agent administration and may reduce AKI and length of stay when employed during cardiac surgery [49, 50]. One study demonstrated that stroke volume optimization—achieved by monitoring the effects of 250 ml^3^ fluid challenges on left ventricle stroke volume—reduced the incidence of AKI following cardiac surgery from 19.9 % to 6.5 % (*P* = 0.002) [51]. The optimal fluid type, vasoactive drug regimen, and guiding hemodynamic parameters for goal-directed therapy are not well established.

Discontinuation of angiotensin converting enzyme inhibitors (ACEi), angiotensin receptor blockers (ARBs), nonsteroidal anti-inflammatory drugs, metformin, and diuretics prior to surgery is typically recommended; however, the largest clinical trial of perioperative ACEi in cardiac surgery patients found higher rates of renal failure, defined as an increase in SCr above 2.5 mg/dl, in patients randomized to placebo compared with ramipril or spironolactone [52], and another clinical trial found evidence that ACEi or ARBs continued until the day of surgery were safe compared with placebo [53]. Some studies indicate that intraoperative calcium channel blocker administration may limit AKI [54–56]. The use of these medications has not gained acceptance, possibly due to concern for vasodilation and reduced cardiac output.

To improve the balance between renal oxygen supply and consumption, physicians have tested treatments that increase blood flow or decrease oxygen consumption. Fenoldopam dilates renal vasculature and therefore increases perfusion. The evidence supporting its use is mixed. Some studies have demonstrated that intraoperative fenoldopam may reduce AKI following cardiac surgery [57, 58], while another showed no benefit and revealed a higher rate of hypotension [59]. These mixed results may be explained by variable effects of fenoldopam on the two distinct renal capillary beds. If glomerular perfusion increases to a greater extent than tubule perfusion, increased solute delivery to the tubules may induce medullary ischemia because increased solute reabsorption requires more oxygen consumption [60]. Dopamine activates D1 receptors like fenoldopam but also D2 receptors. Dopamine administration does not prevent AKI associated with cardiac surgery [61, 62].

Sodium bicarbonate administration also decreases renal oxygen consumption but has not been demonstrated to consistently prevent AKI after cardiac surgery. One study reported a reduction in the incidence of severe AKI (odds ratio (OR), 0.45 (99 % CI: 0.43–0.48); *P* < 0.001) and the need for renal replacement therapy (RRT) (OR, 0.38 (99 % CI: 0.25–0.58); *P* < 0.001) [63], while another showed no effect on AKI (RR, 0.99 (95 % CI: 0.78–1.24); *P* = 0.91) but increased the ICU length of stay for patients who received sodium bicarbonate (weighted mean difference between groups, 2.06 days (95 % CI: 0.54–3.58); *P* = 0.008) [64]. The routine administration of sodium bicarbonate during cardiac surgery is not recommended to reduce AKI [65].

Intravenous acetaminophen reduced plasma concentrations of isofurans, in-vivo biomarkers of oxidative stress, in both adult and pediatric cardiac surgery clinical trials but did not affect AKI [66, 67]. Perioperative dexmedetomidine administration has been associated with a lower incidence of AKI following valvular heart surgery (OR, 0.331 (95 % CI: 0.164–0.667); *P* = 0.002) and a decreased likelihood of progression to a more advanced stage of AKI (OR, 0.307 (95 % CI: 0.152–0.620); *P* = 0.001) [68]. This association may be related to the vasoactive or anti-inflammatory properties of dexmedetomidine and its effects on circulating levels of bone morphogenetic protein 7 (BMP7) [69, 70]. Propofol also increases circulating levels of BMP7, and in mice decreased septic AKI [71]. Other anti-inflammatory regimens have been ineffective. Two recent large multicenter randomized clinical trials of perioperative steroid administration (the DECS trial and the SIRS trial) did not find data to support the routine use of high-dose corticosteroids to reduce AKI following cardiac surgery, although there may be benefit in patients younger than 65 years of age and those with severe (stage 5) CKD [72, 73].

RIPC induces brief transient episodes of ischemia at a site remote from vital organs (e.g., the arm or leg) before organs are exposed to prolonged periods of ischemia and reperfusion. RIPC is hypothesized to attenuate renal damage by inducing the release of signaling molecules in the circulation that activate Toll-like receptors in the proximal tubule epithelia, conditioning the epithelium to tolerate a subsequent inflammatory or ischemic stress [74, 75]. Until recently, studies on the effects of RIPC on AKI in cardiac surgery were small single-center randomized trials that produced conflicting results [76–78]. Three recent multicenter randomized trials were completed to further test the hypothesis that RIPC decreases AKI following cardiac surgery.

Zarbock et al. recruited 240 cardiac surgery patients from four medical centers in Germany at high risk for AKI (Cleveland Clinic score ≥ 6) and randomized them to RIPC or sham treatment. The RIPC treatment group had decreased AKI, RRT, duration of ICU stay, and urine concentrations of IGFBP-7 and TEMP2 compared with the sham treatment group [79]. Shortly thereafter, Meybohm et al. [5] reported in a trial of 1385 patients that RIPC had no effect on moderate or severe AKI compared with sham treatment (6.1 % RIPC versus 5.1 % sham, *P* = 0.45). The study was also a multicenter, double-blind randomized clinical trial in patients undergoing elective cardiac surgery; however, in these patients total intravenous anesthesia with propofol was provided throughout surgery. This practice limits the generalizability of the results, and in addition previous studies have suggested that propofol blunts the protective effects of RIPC on myocardial damage when compared with isoflurane [80]. A third multicenter randomized clinical trial of 1612 cardiac surgery patients performed by Hausenloy et al. found no effect of RIPC on stage 1, 2, or 3 AKI compared with control treatment [81]. So although RIPC treatment is supported by solid preclinical studies, recent large randomized clinical trials in patients undergoing cardiac surgery do not support its use as a preventative measure for AKI.

OpCAB was developed in order to avoid the potential harm of CPB during myocardial revascularization. Clinical trials have not clearly demonstrated that OpCAB surgery reduces the major morbidities associated with cardiac surgery, although OpCAB may lead to marginally lower rates of AKI compared with on-pump surgery [82, 83]. Transcatheter aortic valve replacement (TAVR) might reduce AKI compared with on-pump aortic valve replacement (AVR), since hemodynamics may be more stable during TAVR compared with AVR [84] and systemic inflammation may be lower [85]. The first large randomized trial of TAVR versus AVR found no difference in AKI defined as postoperative SCr > 3.0 mg/dl or initiation of RRT [86].

### Treatment

Treatment options for AKI have focused on attenuating ischemia, reducing intrarenal inflammation, and supportive care. Nutrition is an important component of perioperative care and should not be neglected. Malnourished patients with AKI have an increased risk of mortality [87]. Goals to provide 20–30 kcal/kg/day in patients with any stage of AKI should be met with either parenteral or enteral feeding [6]. If AKI is severe enough to require RRT, higher caloric intake via protein supplementation may be necessary [88]. Glycemic control is also indicated in patients developing AKI [89]. Maintaining a glucose concentration ≤ 150 mg/dl is an appropriate target, while avoiding hypoglycemia (≤80 mg/dl).

In severe AKI, RRT is frequently required to treat hyperkalemia, remove excess fluid, treat uremia, or reverse acidosis. Clinical trials of early versus late initiation of RRT in cardiac surgery patients with severe AKI suggest early RRT may lower mortality and shorten the ICU length of stay [90]. In one study, early RRT was associated with lower mortality (51.5 versus 77.9 %, *P* = 0.001) and decreased time on the ventilator (12.8 versus 18.9 days, *P* = 0.03) [91]. Studies of dialysis dose, frequency, and modality (continuous versus intermittent) have also failed to demonstrate a consistent benefit of one technique versus another. The undisputed benefit of continuous versus intermittent RRT is hemodynamic stability, but otherwise, if a patient meets dialysis criteria (i.e., electrolyte abnormality, acidosis, uremic sequelae, or volume overload), modality does not appear to affect outcomes.

Mesenchymal stem cells (MSCs) possess anti-inflammatory and immunoregulatory characteristics that promote cell survival and tissue repair, and therefore present an attractive potential treatment for AKI [92, 93]. Murine models of ischemic AKI have yielded positive results. Mice that received subcapsular renal injection of MSCs from human exfoliated deciduous teeth following ischemia had decreased SCr, blood urea nitrogen, and inflammatory cytokine concentrations compared with those injected with vehicle [94]. A murine study of rhabdomyolysis AKI (glycerol injection) showed that human liver stem cells (HLSCs) decreased creatinine, urea, hyaline cast formation, and tubular necrosis while enhancing tubular cell proliferation compared with vehicle [95]. The use of human-induced pluripotent stem cells (hiPSCs) was also found to be a potential therapeutic option for AKI because mice with ischemic AKI benefited from renal subcapsular transplantation of hiPSCs [96]. A trial investigating the use of stem cells in cancer patients for the treatment of cisplatin-induced AKI is ongoing (ClinicalTrials.gov NCT01275612). Stem cells may provide a future therapy for AKI following cardiac surgery.

Another potential treatment for AKI is alkaline phosphatase [97, 98]. Alkaline phosphatase converts the proinflammatory adenosine triphosphate into anti-inflammatory adenosine, and in septic rats attenuated plasma concentrations of TNF-α, IL-6, and IL-8 [99]. A randomized clinical trial of septic patients found that intravenous administration of recombinant alkaline phosphatase increased creatinine clearance from 50 ± 27 (mean ± SEM) ml/minute at baseline to 108 ± 73 ml/minute after treatment in the alkaline phosphatase group versus from 40 ± 37 to 65 ± 30 ml/minute in the placebo group (*P* = 0.01), although this functional benefit did not translate into a decreased need for RRT [100].

## Conclusions

AKI after cardiac surgery is common, although most often mild. The development of any AKI remains a major predictor of adverse outcomes, including progression of CKD. Effective prevention and treatment strategies for AKI after cardiac surgery may be on the horizon, and the discovery, validation, and adaptation of biomarkers of nephron damage may accelerate their development as well as shorten the time for diagnosis. For now, efforts to reduce AKI following cardiac surgery and its influence on patient morbidity are confined to hemodynamic manipulations, close attention to intravenous resuscitation strategies including goal-directed therapy and balanced-salt fluid administration, reduced exposure to CPB, and the identification and mitigation of modifiable risk factors.

## Abbreviations

ACEi, angiotensin converting enzyme inhibitors; AKI, acute kidney injury; ARBs, angiotensin receptor blockers; AVR, aortic valve replacement; BMP7, bone morphogenetic protein 7; CKD, chronic kidney disease; CPB, cardiopulmonary bypass; hiPSC, human induced pluripotent stem cell; HLSC, human liver stem cell; IGFBP, insulin-like growth factor–binding protein; KDIGO, Kidney Disease Improving Global Outcomes; MAKE, Major Adverse Kidney Events; MSC, mesenchymal stem cell; OpCAB, off-pump coronary artery bypass surgery; OR, odds ratio; RIPC, remote ischemic preconditioning; RR, relative risk; RRT, renal replacement therapy; SCr, serum creatinine; TAVR, transcatheter aortic valve replacement; TEMP, tissue inhibitor of metalloproteinases.
